# A Model of Filiform Hair Distribution on the Cricket Cercus

**DOI:** 10.1371/journal.pone.0046588

**Published:** 2012-10-04

**Authors:** Jeffrey J. Heys, Prathish K. Rajaraman, Tomas Gedeon, John P. Miller

**Affiliations:** 1 Center for Computational Biology, Montana State University, Bozeman, Montana, United States of America; 2 Chemical and Biological Engineering, Montana State University, Bozeman, Montana, United States of America; 3 Department of Mathematical Sciences, Montana State University, Bozeman, Montana, United States of America; Ecole Normale Supérieure de Lyon, France

## Abstract

Crickets and other orthopteran insects sense air currents with a pair of abdominal appendages resembling antennae, called cerci. Each cercus in the common house cricket *Acheta domesticus* is covered with between 500 to 750 filiform mechanosensory hairs. The distribution of the hairs on the cerci, as well as the global patterns of their movement axes, are very stereotypical across different animals in this species, and the development of this system has been studied extensively. Although hypotheses regarding the mechanisms underlying pattern development of the hair array have been proposed in previous studies, no quantitative modeling studies have been published that test these hypotheses. We demonstrate that several aspects of the global pattern of mechanosensory hairs can be predicted with considerable accuracy using a simple model based on two independent morphogen systems. One system constrains inter-hair spacing, and the second system determines the directional movement axes of the hairs.

## Introduction

**Figure 1:**
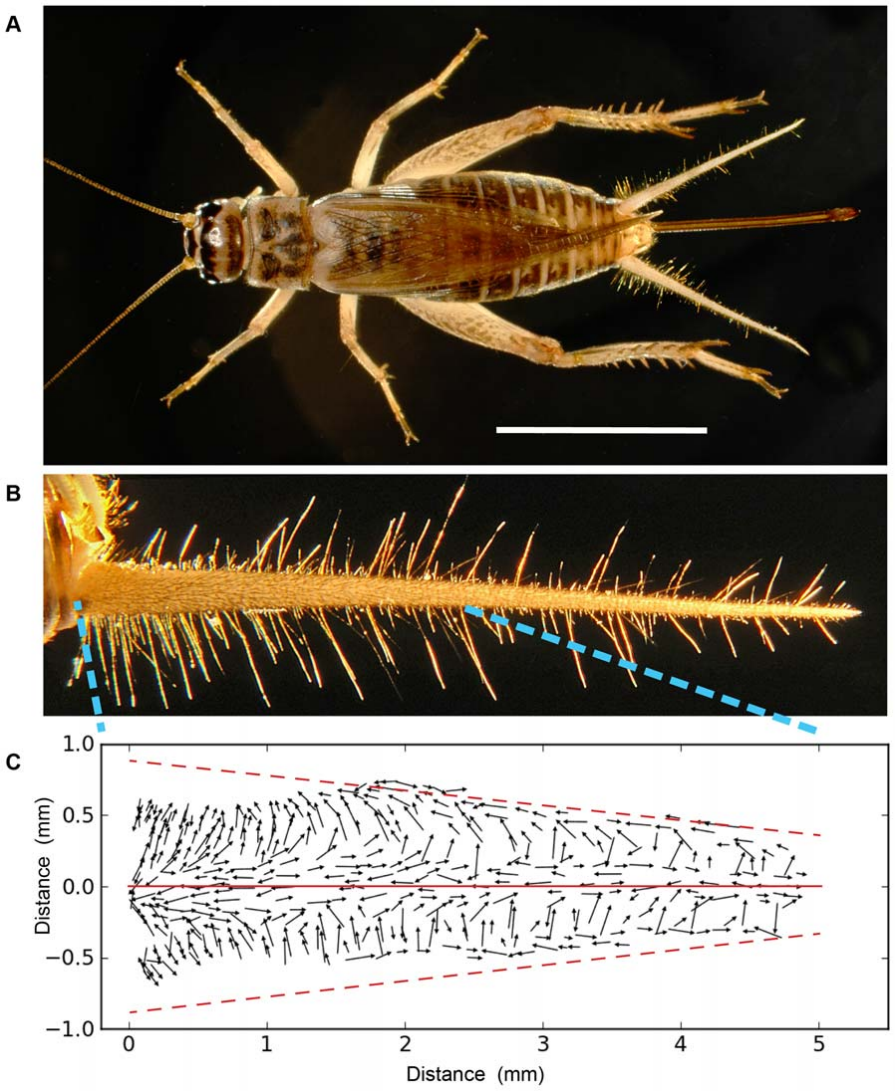
Filiform mechanosensory hairs on the cerci of *Acheta domesticus*. A: An adult female *Acheta domesticus* cricket. Scale bar is 1 cm. The cerci are the two antenna-like appendages extending from the rear of the abdomen. B: An enlarged view of a single cercus. Total length of the cercus is 1 cm. Each cercus is covered with approximately 500–750 filiform mechanosensory hairs, which can be seen in this image like bristles on a bottle brush. C: The locations and excitatory movement directions of all filiform hairs on the basal 50% of the right cercus of a typical cricket. This conical segment of the cercus had been cut along its long axis and flattened out onto a plane. All data is from our previous study [2]. The X and Y axis labels of the bounding box are in millimeters. The image has been oriented within the box so that the lateral longitudinal axis of the cercus is defined as the *X* axis, indicated with a solid red line. That axis corresponds to the *lateral lineage restriction line* used as one of the sources for morphogen M in the model. The origin at X = 0 corresponds to the base of the cercus at its point of attachment to the abdomen. The medial longitudinal axis is indicated with the dashed red lines near the top and bottom edges of the filet preparation: these lines both represent the same axis, and would wrap around to superimpose on one another to form the conical segment of the cercus. That (fused) dashed axis would correspond to the *medial lineage restriction line* used as the other source for morphogen M in the model. The 300 arrows in the plot correspond to the array of mechanosensory hairs observed in this section of the cercus. Each arrow corresponds to anatomical measurements from a single hair socket. The center point of each vector corresponds to the location of the corresponding filiform hair socket, and the length of the vector is proportional to the length of the hair. The direction of each arrow indicates the hair’s excitatory direction of hair movement along its movement plane.

We present the results of a computational modeling study of a simple sensory structure, motivated by a consideration of the developmental constraints on that structure’s functional characteristics. The system we studied is the cercal sensory system of the cricket *Acheta domesticus*. This mechanosensory system mediates the detection, identification and localization of air current signals generated by predators, mates and competitors. On the basis of the information captured by this sensory system, the animal must make decisions rapidly and reliably that are critical for its survival. The sensory apparatus for this system consists of a pair of antenna-like *cerci* at the rear of the cricket’s abdomen (Figure 1). In the adult cricket, each cercus is approximately 1 cm in length. Each cercus is covered with between 500 and 750 filiform mechanosensory hairs, ranging in length from 50 microns to almost 2 mm [1]. These filiform hairs are extremely sensitive to air currents, and the deflection of a hair by air currents modulates the activity of an associated receptor neuron at the base of the hair. All of the information that the cercal system extracts from air currents is derived from the ensemble activity patterns of the combined array of 1000–1500 hair receptors distributed on both cerci. The biomechanical characteristics of the receptor organs for this system may therefore have been a target for natural selection, and may reflect some degree of functional optimization. The general goal of our studies was to determine the simplest developmental model (i.e., a model with the fewest number of free parameters) that is capable of capturing several specific aspects of the global structural organization of the receptor array that were observed in a previous study [2], and which are thought to be of considerable functional importance.

The response characteristics of this mechanoreceptor array are determined by a) the bio-mechanical characteristics of the filiform hairs, and b) the distribution pattern of those hairs on the cerci [2], [3]. It is the second of these two structural characteristics - the distribution pattern of the filiform hairs’ directional selectivities and packing densities on the cerci - that we studied for the analysis presented here. The sensitivity of the sensor array to the direction of air currents emerges from this global aspect of the system’s structural organization. Each mechanosensory hair is constrained to move back and forth through a single plane by a bio-mechanical hinge at the base of the hair. Movement of a hair in one direction along that plane leads to excitation of the associated mechanosensory receptor neuron, and movement in the opposite direction inhibits the neuron [4]. Different hairs have different vectors of motion, and all of those movement vectors are stimulated by air movements in the horizontal plane. The global ensemble of hairs covers all possible directions in the horizontal plane, though the distribution of movement vectors is non-uniform across directions [1], [3], [5], [6], [7], [8], [9], [10]. The differential directional selectivity of the hairs in the cercal array is extremely important from a functional standpoint: air currents from different directions elicit different patterns of activation across the whole ensemble of the cercal hairs, which in turn enables the cricket to discriminate different stimulus directions. Additional studies indicate that the density of the hairs near the base of the cerci is high enough that they might interact with one another through their fluid dynamical environment. In particular, the stimulus threshold for any individual hair is likely to depend upon its proximity to other surrounding hairs, and to the movement axes of those hairs [11], [12], [13].

In this study, we considered four aspects of the structural organization of the array of receptor hairs that have been shown to display low inter-animal variance. Specifically, a) the hairs are organized into bands of uniform movement directionality along the long axis of the cerci, b) there is a systematic rotation in the movement directions of the hairs around the circumference of each cercus, c) there is a non-uniform distribution of hair densities along the length of the cercus, with greater density nearer the base than near the tip, and d) there is a slightly higher density of hairs on the ventral surface of the cercus than on its dorsal surface [2], [3], [6], [7], [8], [9], [10]. These highly-conserved global features of the filiform hair array may be the basis for a substantially increased sensitivity of the system over other possible arrangements with different global organization patterns. The fact that these characteristics are highly preserved across animals, and that they are important determinants of the cercal stimulus sensitivity, suggest that they would be susceptible to selective pressure and subject to control during development.

The general questions that motivated our analysis were as follows: Can these salient aspects of the distribution of hair densities and directional movement vectors be captured with a model based on the current understanding of how morphogen systems function? If so, what is the simplest model that can capture these complex patterns? All data used for this study were drawn from our detailed study of the functional anatomy of the filiform mechanosensory hair array on the cerci of *Acheta domesticus* [2].

The specific goal of the study presented here was to develop the simplest quantitative model for the development of the distribution and directional alignments of filiform hairs, consistent with the observed patterns and with the hypotheses proposed in the earlier developmental studies [1], [2], [3], [6], [14]. The biological basis for the model lies in the body of research by Rod Murphey and his colleagues [3], [8], [9], [14], [15], [16], [17]. Studies of the development of the cercal sensory system indicate that each cercus is divided into two compartments by lines of lineage restriction. One line lies along the medial surface and one lies along the lateral surface [3], thereby dividing each cercus into dorsal and ventral compartments. Studies which analyzed the results of transplanting small strips of cercal epidermis from one animal to another indicated that the two lines of lineage restriction may be serving as sources or sinks for some diffusible morphogen signal that functions to organize the global pattern of hair movement axes. Specifically, mechanosensory hairs along the medial and lateral lineage restriction lines are thought to be constrained to move in the longitudinal direction (*i.e.*, along the long axis of the cercus), whereas hairs located away from the compartmental boundaries are thought to be constrained to move along directions that are oblique to the long axis. This general hypothesis was the basis for our model.

We modeled a segment of the surface of a cone, corresponding to an equivalent segment of the surface of a cercus. The model was developed with the goal of predicting: (a) the sites at which hair sockets would be induced on a cercus, and (b) the alignment of the movement axis for the hair associated with each of these sockets. We hypothesized that the location of the hairs is determined by developmental processes mediated by two different morphogens which diffuse throughout the 2-dimensional conical sheet, and which interact with receptors through a first-order reaction. One set of equations in the model was associated with a morphogen gradient that determines the movement vector of each hair. Specifically, the direction of motion of each hair was influenced by the distance of the hair socket from the lines of lineage restriction. The second set of equations was associated with a morphogen that determines inter-hair spacing. The morphogen mediating this patterning is assumed to originate at the base of each hair socket. This simple model yielded remarkably good replication of the pattern of the cercal filiform mechanoreceptor hair array. We note that the model was developed at a general level, and does not stipulate the chemical natures of the morphogens, nor the specific mechanisms through which their gradients are established or interpreted by target cells.

Our model was based on a linear combination of solutions to two linear diffusion problems to create a patterned array of hairs. However, there are important differences between our computational approach and the conventional Turing “reaction-diffusion” (RD) model used for similar purposes in previous studies [18], [19], [20], [21], [22], [23]. First, our model was discrete and stochastic, and solved through the minimization of a cost function using a Monte Carlo algorithm [24], whereas Turing models are continuous and deterministic. Second, Turing models require two morphogens (a short-range activator and a long-range inhibitor) to generate an array having a specific number of hairs with a specific inter-hair spacing. The spacing in a Turing model is determined by the interplay between the reaction parameters and the diffusion rates of the two morphogens, which, together with the size of the domain, determines the total number of hairs that will be generated and the distribution pattern within the domain. Our calculations only required a single morphogen (an inhibitor that originates from each hair or hair socket) to specify inter-hair spacing, because the model was initialized with a fixed number of hairs already existing within the domain. The role played by the activator morphogen in a conventional Turing RD model was subsumed into our model as an initialization parameter.

## Methods

### Inter-hair Spacing: Morphogen S

For the model, the first morphogen (defined as morphogen S, indicating inter-hair *Spacing*) is treated as a diffusive chemical signal that is produced at each hair socket and is subsequently degraded through a first-order reaction. This first morphogen is hypothesized to inhibit the filiform hairs from being located near one another. As indicated earlier, our modeling approach does not invoke a second “activator” morphogen as is typically the case for Turing RD models. As described in more detail below, the function played by the activator morphogen in a conventional Turing RD model is subsumed into our model as an initialization parameter: we seed the simulated domain with a fixed number of hairs in a random pattern, and re-position the hairs under the influence of the inhibitory morphogen S.

There are many examples of similar signaling mechanisms in biology (see, for example, [21], [22], and recent reviews [23], [25]). Chemical diffusion and first-order decay imply that the strength of the chemical signal decays exponentially with distance away from the source [26], 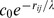, where 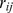
is the distance from the hair socket generating the signal to other hairs, c_0_ is the concentration at the socket (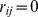), and λ is the characteristic decay distance. Since this chemical is believed to inhibit the growth of new hairs near existing hairs, we express this cost function mathematically as being proportional to the normalized concentration, 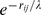. Palka et al. observed that filiform hair density is highest at the base of the cercus and lowest near the tip [5], and the hair density gradients on three cerci were recently measured quantitatively [2]. Parameters in our model were set to match these measurements: the decay distance, λ, was set to 0.2 mm at the base and was set to increase linearly to 0.4 mm at the end of the model domain near the cercus tip. The characteristic decay distance was chosen to be approximately half the distance between filiform hairs.

It has been suggested that this simple diffusion and linear, first-order degradation model is not as robust as an alternative model that has diffusion and nonlinear, second-order degradation [27], [28]. For the case of second-order degradation, the chemical signal decays at with a power law rate and is proportional to 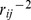. We have chosen to use the simpler and more common first-order decay law, but we also tested a second-order degradation law in the model described below and noted that the predictions, after scaling to fit the experimental data, were similar (results not shown).

### Hair Movement Direction: Morphogen M

The second morphogen (defined as morphogen M, indicating hair *Movement* direction) is treated as a diffusive chemical signal that is produced along the medial and lateral lineage restriction lines [3] corresponding to the solid and dashed lines in Figure 1C, respectively, and is hypothesized to account for the organization of the hairs into bands of uniform movement directionality along the long axis of the cercus. Note that for our model the same morphogen M is released from both restriction lines. As was the case for morphogen S, the concentration of this second morphogen M is also assumed to be governed by steady-state diffusion with first-order decay. As a result, its concentration decays with distance away from the source, according to the relation 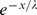 where x is the distance to the signaling line. In order to describe the action of this morphogen in mathematical terms, we use the following form for its concentration:
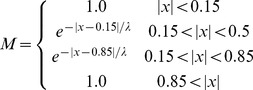
(1)where x is the fractional distance from the medial lineage restriction line to the lateral restriction line (i.e., x = 0 at the medial line and x = ±1 at the lateral restriction line). This morphogen does not determine which direction along the movement plane is excitatory and which is inhibitory, but only the directionality of the underlying cuticular hinge mechanism. For example, at high concentrations hairs must be close to 0, +180, or −180 degrees. At low concentrations, hairs must be close to ±90 degrees, using the directional format defined in Figure 1C.

### Cost Function for the Model

We derived a cost function to capture the salient features of the mechanosensor array described in earlier studies. Considering all of these observations and model assumptions, and using the normalized concentration for M derived in eq. 1, the cost function for this signaling molecule can be written as 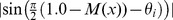 for 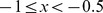 or 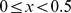 and the cost function is 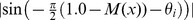 for 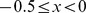 or 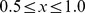. This cost function is based on the angle difference between the hair movement direction, θ_i_, and the angle prescribed by the morphogen concentration, 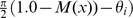. The sine is taken of the angle difference so that 1) angle differences of +/−180 degrees are not penalized, and 2) the cost function has a maximum size of 1. To account for the increased density of hairs on the ventral surface, a small correction factor, a*, was applied to the cost function for the hair deflection angle. Specifically, the cost function value was reduced by 10% (i.e., a* = 0.1) for hairs aligned at a positive angle and located on the upper half of the domain. This resulted in a ratio of hair densities in the ventral hemi-cone vs. the dorsal hemi-cone of 1.22, which matched the experimental observations [2].

Combing these mechanisms together into a single function and introducing the parameter, c_1_, to account for the difference in strength between the two short range, hair-to-hair interactions and the one long range signal from the lateral lineage restriction lines, leads to the following equation for hair i:
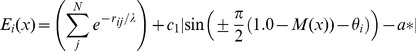
(2)where E_i_ is the value of the cost function for a single hair and r_ij_ is the distance between hairs i and j. The complete cost function for all hairs is then
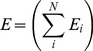
(3)for a given distribution of hairs on the cercus.

The cercus is shaped like a cone, and when measuring distances between two points on the surface of the cone, it is important to use the geodesic distance between the two points. We approximated the geodesic distance numerically, because the analytical solution requires solving a system of non-linear equations. The numerical approximation of the geodesic distance depends on the number of discrete steps between the two points, and, after extensive testing to ensure at least 2 digits of accuracy, 100 discrete steps were used for our simulations. It is possible that the rate of diffusion varies with direction and thus diffusion is anisotropic, but the experimental hair distribution patterns do not indicate a strong anisotropy and we wished to avoid introducing another unknown parameter into the model.

The following algorithm was used to find a distribution of hairs that approximately minimized the value of E. All models were set to contain 300 hairs, which approximates the average number determined from quantitative measurements [2] in real cerci. The hairs were initially distributed randomly throughout the domain and assigned a random direction of motion. In all cases, a uniform random distribution was used. Once the domain and initial conditions had been set, an initial value for the function E was calculated using equation (3). A randomly selected hair was then translated to a new, randomly selected location, and randomly rotated by some angle. The change in the cost function E based on this move was determined. If the function increased, the move was not allowed and the hair was returned to its previous position and angle, but, if the function decreased, the move was allowed and the value of the function was updated.

This process of randomly moving hairs was repeated for P iterations. If P is set to a small value (e.g., P = 1000) the original random distribution and alignment is largely preserved, and the prediction is not similar to experimental observation. If P is set to a large value (e.g., P = 10^8^) almost all of the random structure is lost, the hairs are nearly uniformly distributed, and the hair alignment directions translate smoothly between hairs moving parallel to the cercus axis and hairs moving perpendicular to the cercus axis. This operation yielded a uniform, nearly perfectly optimized distribution. This near-perfect optimization was assessed against the result of solving the Turing reaction-diffusion system for this data set, which yielded a single, optimal solution (results not shown). However, morphogen production and diffusion are often subject to a considerable level of noise. The choice of P can be viewed as a regulation of the level of noise preserved in the model prediction. For most of the results shown below, P is set to 10^5^ so that some of the random variation is preserved. Justification for the choice of P = 10^5^ and the effects of choosing other values are examined in the Results section.

### Assessment of model performance

For our study, one parameter of interest was the inter-hair spacing between the sensory hairs in the array. We used spatial autocorrelation analysis to assess the extent to which the global spatial arrangement of sensors generated by the model matched the observed spacing pattern in real animals. The metric we employed was Ripley’s L function, which quantifies the degree of randomness in the spatial pattern of points for various sizes of a circular search window. Spatial randomness can be tested by varying the size of the circular search window and counting the number of observations within the window. Ripley’s L function is calculated as
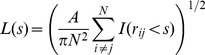
(4)where A is the area of the region containing the points, N is the number of points (for our case, the number of hairs on the actual or model cercus, which was approximately 300), s is the radius of the search window, and 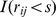 is 1 if 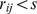 and 0 otherwise. Conceptually, the procedure was as follows: we would pick one of the 300 hairs on a cercus sample (either an actual cercus or a model-generated pattern), center a small circular window (with a radius of s) on that hair, and count the number of hairs within that window. We would then shift the center of that measurement window to one of the other 300 hairs, and count the number of hairs. The L value would be calculated for that window size over all 300 hair locations, and plotted as a single data point. We would then increase the radius of the window, and re-calculate L around all hairs for that larger window. This calculation would be reiterated over ever-increasing window diameters. For a homogeneous Poisson process, L(s)≈s, and a plot of L vs. s is approximately a diagonal line. In other words, a totally random placement of the hairs within the surface of the cercus would yield a spatial distribution that included some very closely-clumped groups, some very widely-spaced groups (i.e., leaving big open patches without hairs), and a range of intermediate spacing values, yielding an overall plot that would range linearly between zero (for a very small window) to a large value for a large diameter window. Deviations from the diagonal line can be used to construct a test for complete spatial randomness. Function values above the diagonal line in the plot indicate spatial clustering (i.e., points that are clumped together in space), and values below the diagonal line indicate spatial segregation or points that are dispersed with some uniformity. The significance of deviations from the diagonal line is highly dependent upon the application, but deviations of more than 5–10% are typically considered significant [29].

## Results

We modeled the basal half of a typical adult cercus, i.e., a segment of the cercus that started where it emerged from the posterior end of the abdomen and extended to a distance of 5.2 mm (a typical adult cercus is approximately 1 cm in length). The diameter of the conical segment was 0.54 mm at the base and decreased to 0.26 mm at the end of the 5.2 mm segment. Our model was based on quantitative anatomical data obtained in our previous study [2].

There were four specific features of the observed distribution of receptor hairs on the cerci that we attempted to capture with our model: a) the hairs are organized into bands of uniform movement directionality along the long axis of the cerci, b) there is a systematic rotation in the movement directions of the hairs around the circumference of each cercus, c) there is a non-uniform distribution of hair densities along the length of the cercus, with greater density nearer the base than near the tip, and d) there is a slightly higher density of hairs on the ventral surface of the cercus than on its dorsal surface (the average ratio of hair densities in the ventral hemi-cone vs. the dorsal hemi-cone is 1.22 [2]). These features can all be observed in Figure 1C. This figure plots the location and direction of excitatory movement for all filiform mechanosensory hairs measured in the basal 50% of one cercus in an earlier study [2]. For this and all other all illustrations in this report, that conical segment is represented as an unrolled, flattened surface, with distance along the *X* axis (5.2 mm) corresponding to position out along the length of the cercus, and position along the *Y* axis corresponding to the circumferential position around the cercus. The domain height tapers linearly from approximately 1.7 mm down to 0.8 mm, which is consistent with the change in cercus circumference for a 5.2 mm segment. The X axis, indicated with a thin solid line, corresponds to the *lateral lineage restriction line* used as one of the sources for morphogen M in the model, as described below. The origin at X = 0 corresponds to the base of the cercus at its point of attachment to the abdomen. The medial longitudinal axis is indicated with the dashed lines near the top and bottom edges of the filet preparation: these lines both represent the same axis, and would wrap around to superimpose on one another to form the conical segment of the cercus. That dashed axis corresponds to the *medial lineage restriction line* used as the other source for morphogen M in the model, as described below. The 300 arrows in the plot correspond to the array of mechanosensory hairs observed in this section of the cercus. Each arrow corresponds to anatomical measurements from a single hair socket. The center point of each vector corresponds to the location of the corresponding filiform hair socket, and the length of the vector is proportional to the length of the hair. The direction of each arrow indicates the hair’s excitatory direction of hair movement along its movement plane (movement in the opposite direction along this plane would inhibit the receptor). It is clear that the movement planes of hairs in close proximity to one other are very similar, even though the actual direction of activation along the movement planes of nearby hairs may differ by 180 degrees.

Note that the vectors seem to be “scooped away” from the dashed lines corresponding to the medial lineage restriction line within the 1.5 mm region at the base of the cercus. That region has very few filiform hairs, but instead contains the entire array of clavate mechanoreceptors [1]. The clavate sensors mediate sensitivity to gravity and acceleration, and are all localized to this patch on the baso-medial face of the cerci. We did not attempt to model the distribution for the clavate hairs, nor did we attempt to restrict the placement of filiform hairs from this region. Rather, all of our simulations treated this region as a normal extension of the rest of the cercus. Special consideration of this region will be dealt with in future models.

The model we studied has a total of 5 parameters: 1) the total number of hairs in the array, 2) the correction factor a* that accounts for the observed difference in relative density of receptor hairs on the dorsal and ventral cercal surfaces, 3) the characteristic decay distance λ over which the concentrations of morphogens S and M decrease as they diffuse away from their sources at the base of each hair socket and the lateral restriction lines, respectively, 4) the balance in strength between the two signaling mechanisms, c_1_, and 5) the number of iterations P carried out for the Monte Carlo calculations. The first 3 parameters were obtained directly from existing experimental data, leaving the computational model with 2 adjustable parameters: P and c_1_. The parameters P and c_1_ effect the spatial distribution of the hairs and the distribution of their directions of motion. We determined that values of c_1_ = 10 and P = 10^5^ for these parameters yielded predictions as close as possible to the actual data obtained from measurements of cercal filiform arrays.

**Figure 2:**
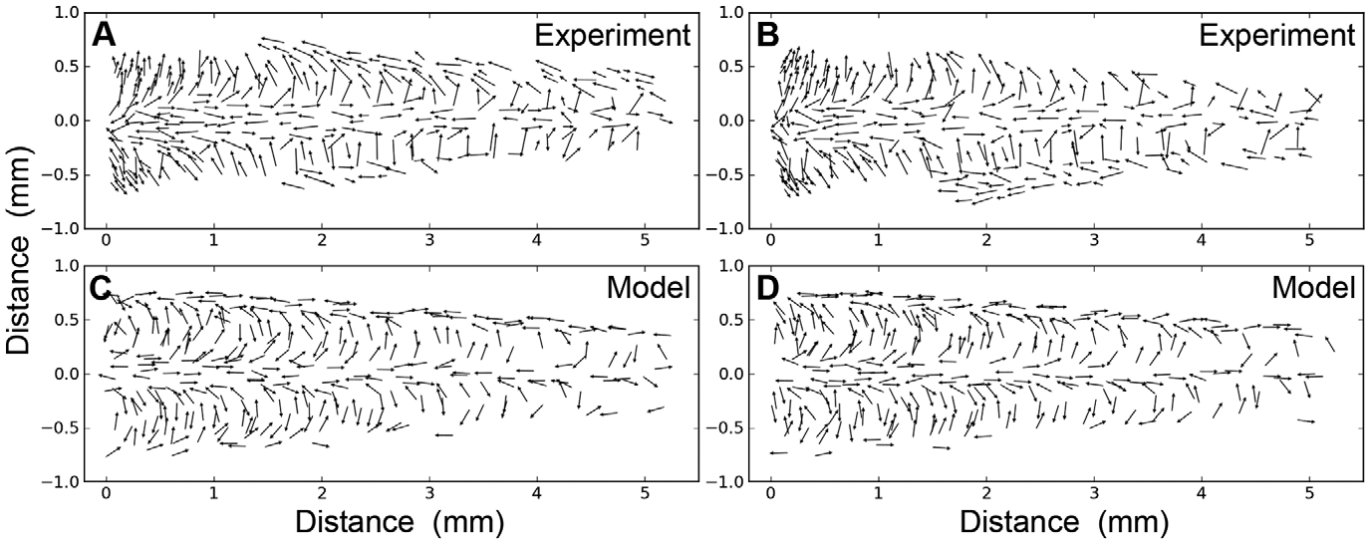
The distribution and movement directions of filiform mechanosensory hairs. A, B: Experimental measurements of filiform hair positions and movement directions in two different cricket preparations. C, D: Model predictions for an equivalent segment of the cercus for two different simulations using different initial hair positions. As indicated in the text, filiform hairs are excluded from a small region near the base of cricket cerci containing the clavate sensor array, corresponding to the “scooped-out” regions in panels A and B. We did not attempt to model those restrictions in our simulations, and so the computed arrays in panels C and D show the vectors extending all the way to the medial restriction lines. All labels on the bounding boxes are in mm.

Two different sets of model results obtained with these parameters are shown in Figure 2C and 2D, to illustrate the intrinsic variability in the simulation protocol. These results can be compared qualitatively to the data from two actual biological specimens in 2A and 2B. Larger values of P result in less variability, and lower values of P result in greater variability. For P = 10^5^, the cost function is very near the minimum value, and the results are similar at a more global level, but there is still some local variability.

In order to obtain a more quantitative comparison of the model predictions to experimental measurements, two different metrics were used. First, the hair movement direction distributions predicted by the model were compared to the experimental data. Second, a spatial autocorrelation function was used to compare the spatial distribution of sensory hairs predicted by the model to those in actual cerci.

### Assessment of the Accuracy of Predictions of the Hair Movement Directions

**Figure 3:**
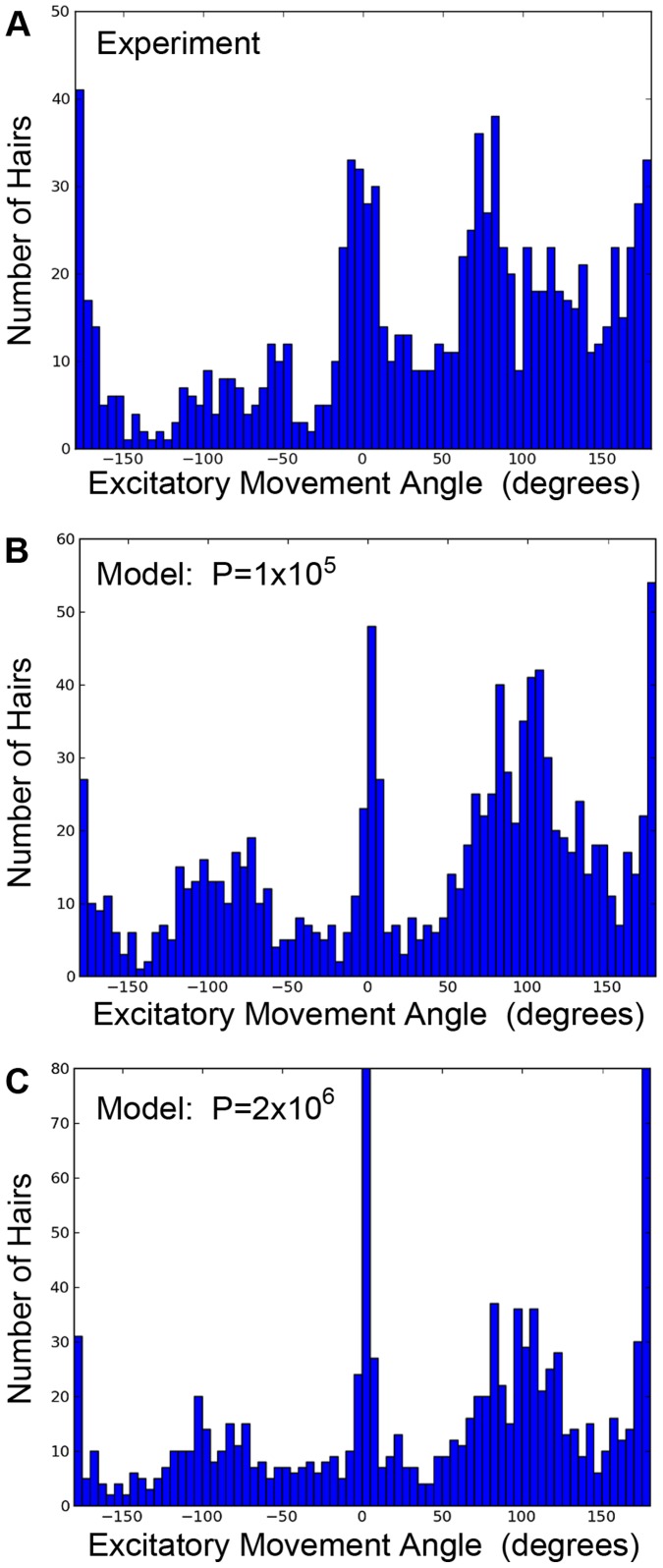
Histograms showing the distribution of hair movement angles. A: Summed distribution for experimental measurements from three cerci. B: Summed distributions for three simulation runs using different initial hair positions, using 1×10^5^ iterations to minimize the functional. C: Summed distributions for three simulation runs using different initial hair positions, using 2×10^6^ iterations to minimize the functional. The agreement between the simulations and the experimental distribution were acceptable for P>10^5^. Note the differences in *Y*-axis scales between the panels.

The first metric used for comparing the model prediction to actual cercal filiform arrays is the distribution of hair movement directions. A comparison of the model and experimental measurements of hair motion directions are summarized in the histograms in Figure 3. For these histograms, each bin corresponds to a 5 degree range in the direction of hair motion, and the height of the bin corresponds to the number of hairs within that range. The very non-uniform distribution of movement directions corresponds to the non-uniform “banding” of the receptor directions observed experimentally in earlier studies, referenced above. Note that in Figure 3 we use a different labeling scheme for the X axis (hair movement direction) than we used in our previous report [2]: for the purposes of this figure, it was simpler to define the direction axis as the long axis of the cercus, rather than to the axis of the animal’s body. The dominant direction of motion observed experimentally (Figure 3A) is parallel to the cercus axis (i.e., here defined as θ_i_ = 0 or ±180°). The model is initialized with a full set of hairs having a uniform, random distribution of directions and locations. As the hairs are re-distributed to minimize the cost function, the directional distribution approaches the distribution observed experimentally, with the most similar distribution being found for P = 10^5^ (Figure 3B). When P>10^6^, the model is near the minimum function value and the distribution of hair motion directions is still similar to the experimental distribution, but the results, overall, show less randomness and individual variability than the experimental results (Figure 3C).

**Figure 4:**
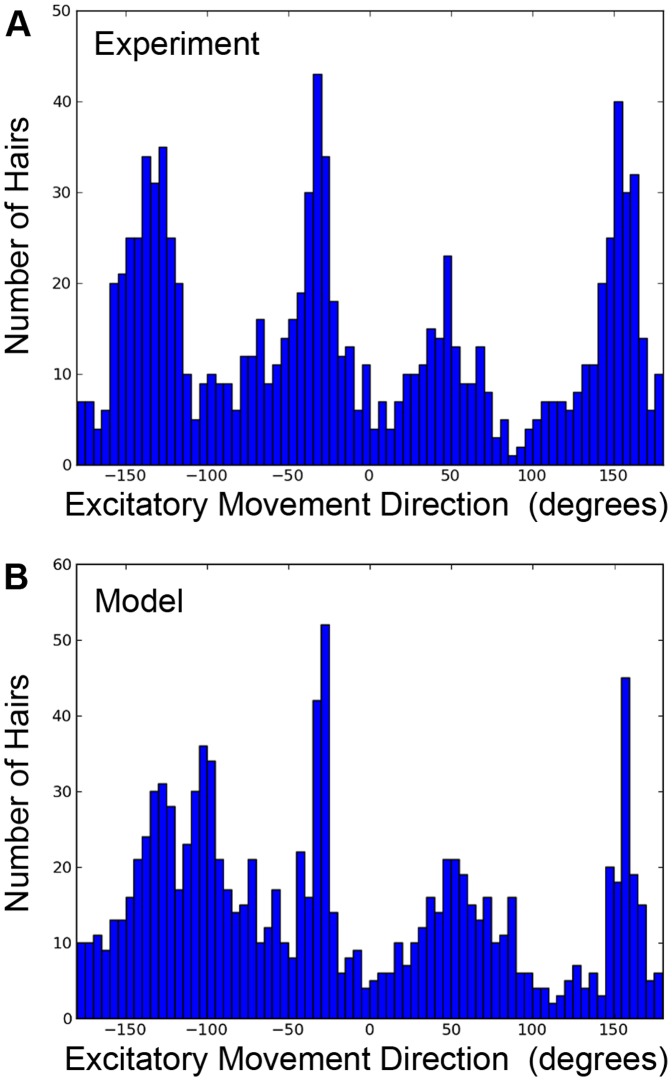
Histograms showing the distribution of hair movement angles transformed into the animal’s coordinate system. 0 degrees corresponds to an air current oriented at the cricket from directly in front. A: Summed distribution for experimental measurements from three cerci. B: Summed distributions for three simulation runs using different initial hair positions, using 1×10^5^ iterations to minimize the functional. Both histograms show four characteristic peaks. Note the differences in *Y*-axis scales between the panels.

The histograms in Figure 3 represent the directions of motion of the hairs as measured directly from the planar surface after cutting and flattening, as in Figures 1C and 2. However, it is straightforward to transform these angles into the true movement directions with respect to the animal’s body. The transformation corrects for wrapping the flattened surface onto a cone and rotating the cercus 30° out counterclockwise from the body axis of the cricket. The directional data histogram shown in Figure 4A is equivalent to that shown Figure 3A, except that it shows the data transformed to this body-centric coordinate system. This enables direct comparison with data published in the report from which the data was extracted [2]. In Figure 4, 0 degrees is defined as a vector pointing directly forward along the main axis of the cricket. In this transformed system, the hairs along the lateral line which were previously characterized as oriented to 0 or ±180 degrees are now aligned at approximately 150 degrees or −30 degrees. Once these transformations are made, the experimentally-observed sensory direction histogram (Figure 4A) shows coverage of the full 360 degrees around the cricket with four distinct peaks. The model predictions for three independent simulations under different initialization patters are shown in Figure 4B. The model results show the same four characteristic peaks, and show a high degree of similarity with the experimental measurements.

### Spatial Autocorrelation Analysis of Inter-hair Spacing

**Figure 5:**
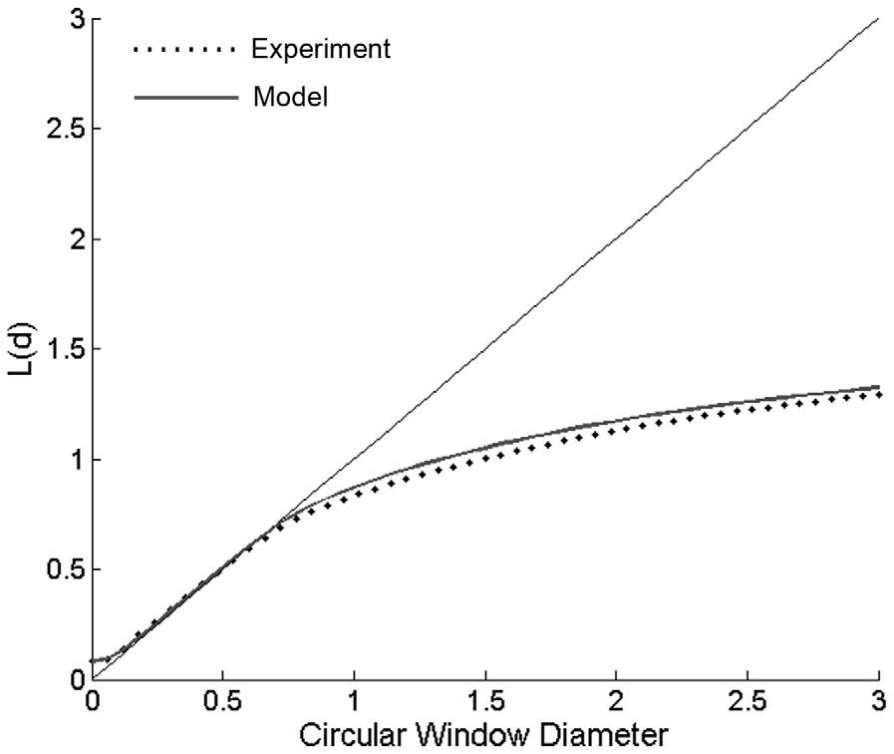
Spatial autocorrelation comparisons. Value of the Ripley L function of the inter-hair spacing for the model hair distribution (solid line) and experimentally measured hair distribution (circular symbols) for different circular window diameters (in mm). A distribution following a Poisson process would fall on the straight thin diagonal line. Values above the diagonal line indicate spatial clustering, and values below the line indicate spatial segregation. For circular window sizes of 1 mm or larger, the model prediction and experimental measurements show spatial segregation for the filiform hairs. The hairs are observed to be more uniformly distributed than would be observed in a random process indicating the existence of some mechanism (hypothesized as morphogen S) that prevents hairs from being located near one another.

In order to assess the extent to which our model’s predictions of the hair spacing pattern matched experimental observations of biological specimens, we used a spatial autocorrelation technique. Spatial autocorrelation refers to the pattern in which observations from nearby locations are more likely to have similar parameter values than by chance alone [30]. There are a number of statistical measures for spatial autocorrelation analysis [30], [31], [32]. As described in detail in the Methods section, the metric we calculated was Ripley’s L function, which quantifies the spatial pattern of points for various sizes of a circular search window. Figure 5 shows Ripley’s L function calculated for the experimental data obtained from one preparation (circular symbols) and model data with c_1_ = 10 (solid line) using the same number of hairs (N = 300) and domain size. A pattern of hairs generated through a totally random Poisson process (i.e., without any constraints on inter-hair spacing) would yield Ripley L values falling on the straight diagonal line. Function values falling above the line would indicate spatial clustering, and values falling below the diagonal line would indicate spatial segregation [31], [33].

We found that for small search windows less than about 0.75 mm in diameter, the pattern of hairs corresponded to what would be expected for a random Poisson process. However, as the size of the search window was increased beyond 0.75 mm, the number of hairs within each circular window deviated from that expected for a spatially random process, and conformed to what is expected for spatial segregation. This plot supports the visual impression that the filiform hairs on this cercus specimen are not randomly located, but have a more uniform distribution than a completely random process would display. This supports the operation of some process that acts to prevent hairs from being located near one another. Note that we do not plot error bars on the plots of experimental or modeled data. This is due partially to the difficulty of choosing a meaningful metric for representing the uncertainty in the calculations, but due largely to the relatively low uncertainty. This issue is considered in detail in the Supplemental Material. An alternative version of Figure 5 showing Ripley’s L function is presented in Supplemental Figure S1, plotting the experimental data from 3 different cerci. Comparison of the plots for 3 different data sets serves as a characterization of variation. As inspection of that supplemental figure demonstrates, the 3 plots are almost superimposed, indicating a high degree of significance.

Comparing the Ripley L function for the model distribution to the experimental hair distribution, we observe strong agreement for all window sizes. Significantly, the model distribution displays the same spatial segregation, i.e., the same uniform spatial distribution, for window sizes that are larger than 1 mm. This result supports the plausibility of the hypothesis that a morphogen could be acting to inhibit the location of hairs close to one another.

As mentioned previously, chemical diffusion rates may be anisotropic in the region of interest, which would primarily impact the spatial distribution of the hairs and not the angular distribution of hairs. Therefore, Ripley’s L function provides a tool to examine the impact of assuming isotropic diffusion rates. Overall, the model predictions are only slightly impacted when the diffusion rates are different in the axial and radial direction. As anisotropy is introduced (i.e., as the search windows are changed from circles to ellipses with particular orientations), the hair spacing becomes more regular in one direction relative to the other, which decreases the value of Ripley’s L function at length scales less than 1 mm. However, the impact was very minor (data not shown). Overall, the experimental data does not support the existence of strong anisotropic diffusion rates, and small anisotropy differences have a much smaller influence on the model results than the two parameters used in the model.

### Sensitivity of the Model to the Numerical Value of the Cost Function

**Figure 6:**
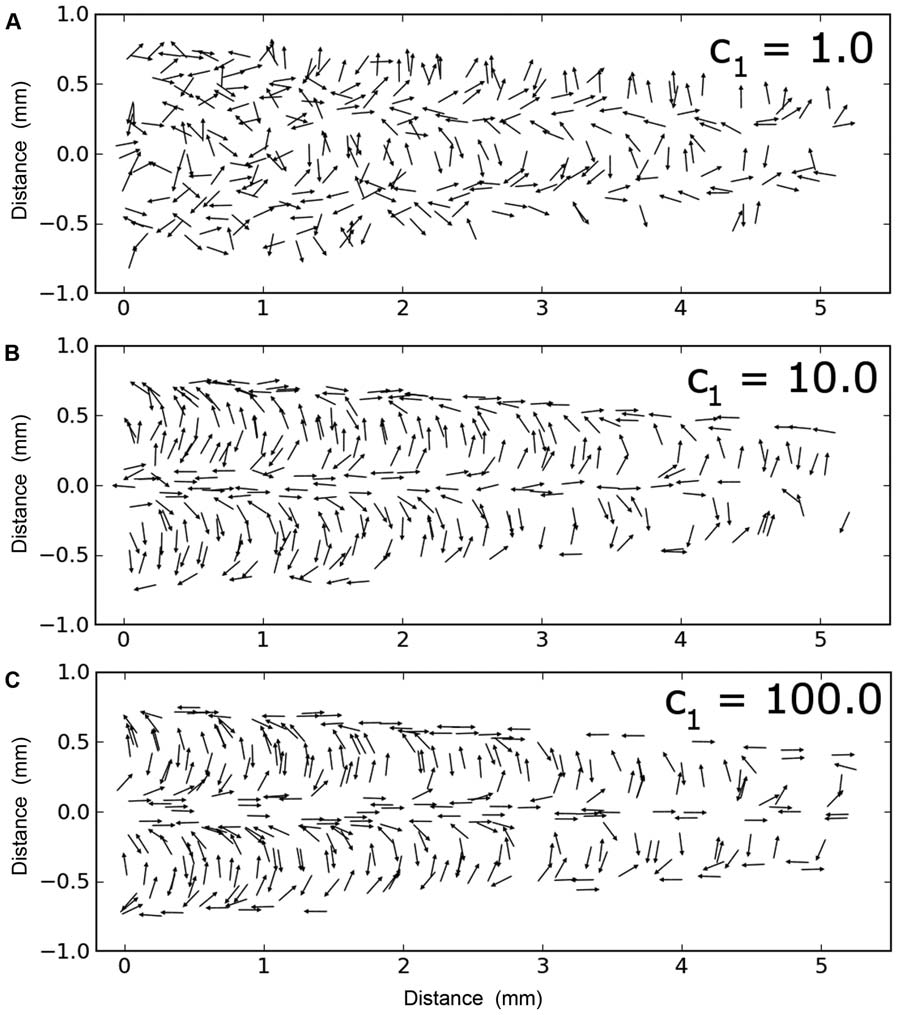
Sensitivity of the distribution of hairs on the value of *c*_1_ used for minimization of the cost function. A. Example of a final configuration obtained with c_1_ = 1. B. Example of a final configuration obtained with c_1_ = 10. C. Example of a final configuration obtained with c_1_ = 100.

Figure 6 illustrates the dependence of the distribution of hairs on the value of ***c***_1_ used for minimization of the cost function. c_1_ controls the relative importance of hair spacing versus direction of motion alignment to the minimization process. With lower values of c_1_ (Figure 6A, c_1_ = 1.0), directional alignment is relatively unimportant in the cost function, and the minimum is achieved when the hairs are distributed in a relatively uniform manner with little regard to alignment. Higher values of c_1_ (Figure 6C, c_1_ = 100) cause the hair alignment term in the function to dominate, and the lack of relative importance of hair spacing leads to yield greater clustering of the hairs. The best agreement between model and experimental data occurs with c_1_ = 10 (Figure 6B). We can understand why this value gives the best agreement by considering the relative sizes of the terms in the cost function. If each hair is surrounded by approximately six other hairs at a distance of roughly 0.08 mm, the hair separation part of the functional will be approximately 6·e^−0.3^ = 4.4. The directional alignment part of the cost function, on the other hand, will be equal to approximately 10·sin(Δθ), where Δθ is the difference between the prescribed and the actual hair alignment angle. If the alignment difference is greater than approximately 5 degrees, this term will dominate in the cost function and the algorithm is likely to find a lower energy state simply by improving the alignment. If the alignment difference is less than 2 degrees, the spatial distribution part of the cost function will dominate and reducing the cost function will require a more uniform distribution. In summary, a value of c_1_ = 10 provides a balance between the dual goals of obtaining a uniform spatial distribution and obtaining proper alignment.

## Discussion

To reiterate the questions that motivated this study: Can the salient aspects of the distribution of hair densities and directional movement vectors be captured with a model based on the current understanding of how morphogen systems function? If so, what is the simplest model that can capture the complex patterns documented in our recent anatomical study? Our general interpretation of the results presented in this report is that a simple model based on concepts proposed by Rod Murphey and his colleagues yields remarkably good replication of the pattern of the cercal filiform mechanoreceptor hair array.

The model is simple, in the sense that only one morphogen is used for each of the two attributes we considered: one system for the determination of hair movement direction, and the other for the inter-hair spacing. The final configuration of the sensor array is calculated as a simple linear combination of the solutions to the two linear diffusion problems. As we noted, our use of only a single morphogen for determination of the inter-hair spacing was a mathematical simplification used to facilitate more efficient computation than with a conventional Turing model: the biological implementation of a conceptually equivalent Turing mechanism for hair spacing would require the involvement of a second morphogen with a unique reaction parameter and diffusion rate. As a demonstration, a full conventional Turing reaction–diffusion model using two morphogens is presented in the supplemental materials (Figure S2, Supplemental Material). Our use of a single morphogen avoids the need for solving multiple partial differential equations to predict the distribution of two morphogens having diffusion rates that differ by an order of magnitude or more. Specifically, our numerical approach is scales as O(N^2^), where N is the number of hairs. Traditional finite element or finite volume approaches to the Turing reaction-diffusion equation typically scale as O(N^4^) or worse for similar geometries (see Supplemental Material). There is, however, a “cost” for this simplification. Even though our calculations are compatible with all proposed biological mechanisms, our current computational approach precludes any straightforward attempt to model the dynamics or specific molecular mechanisms underlying the actual biological developmental processes.

Why do we think that the model’s fit is “remarkable”, since we know from experience that the development of virtually any structure could be simulated to any arbitrary degree of accuracy, given a model with enough free parameters? We consider the fit to be remarkable because such a simple model is capable of capturing such extreme non-uniformity in the hair distribution pattern with such fidelity, under what would seem to be very complex boundary conditions (i.e., a long conical surface with two lines acting as sources and/or sinks). Specifically, the global patterns of the filiform hairs’ directional selectivities and packing densities on the cerci display remarkably complex structure: the distributions of preferred hair directions on the two cerci result in a very non-uniform distribution containing multiple distinct peaks, as shown in figures 1C, 2A, 2B, 3A and 4A. Yet the fit obtained with this simple model captures the longitudinal strips of hairs, centered at the appropriate stimulus angles, and progressing systematically through all angles. Further, the spatial autocorrelation metric we used for assessing the inter-hair spacing (the Ripley L function, shown in Figure 5) showed the model and actual distributions to be statistically indistinguishable across all length scales.

The success of such a simple model is significant within the context of hypotheses concerning the functional optimality of sensory systems. It is a common assumption that any features of sensory structures that show low inter-animal variability (such as the pattern of the cercal filiform afferent array) may be under constraints imposed by functional effectiveness, energetics, developmental processes and/or phylogenetic legacy. However, it is extremely difficult to assess the relative weighting of these different constraints. In a previous report, we hypothesized that the pattern of filiform hairs may approach an optimal configuration with respect to function constraints imposed by the fluid dynamics of air flow over this sensory organ [2]. Previous studies of the biomechanical properties of the filiform receptor hairs in this and similar species demonstrate that the density of the hairs is high enough that they interact with one another through fluid dynamical coupling [11], [12], [13], [34]. In particular, the stimulus threshold for an individual hair depends upon its proximity to other surrounding hairs, and to the movement axes of those hairs. Thus, the highly-conserved features of the filiform hair array pattern were hypothesized to be the basis for a substantially increased sensitivity of the system over other possible arrangements with different organization patterns; i.e., the array pattern might correspond to the global functional optimum with respect to one or more functional criteria like absolute threshold, signal-to-noise ratio, or multi-dimensional feature detection. This same argument about functional optimization might logically be extended to other systems of mechanosensory hairs in this species: Murphey and colleagues demonstrated that cercal clavate hairs in *Acheta domesticus* are uniquely identifiable based on position and birthday [14], [35], [36]. In fact, there has been a long history of studies of re-identifiable sensory hairs in insects and arthropods dating back to foundational work carried out in Drosophila by Curt Stern (see [37] for a beautifully illustrated review and definition of important questions and hypotheses related to the genetic and developmental mechanisms underlying the positioning of the bristles). Since that early work, the notion has been discussed that the low variability in these patterns may reflect some degree of optimization through natural selection. More recently, Bathelier et al. presented data showing near-maximal mechanical efficiency of cricket cercal filiform hairs within the stimulus frequency range that is of the greatest behavioral significance to the animal’s survival [38].

However, the results of the simulations presented here indicate that the simplest possible conceptual model is capable of generating the spatial pattern; i.e., no “higher-order tweaking” of developmental mechanisms is required beyond the linear interaction of two extremely simple morphogen systems. Thus, a perfectly reasonable alternative hypothesis to explain the low inter-animal variance in the receptor array pattern is that the morphogen-based developmental mechanisms are the primary (and robust) determinants of the hair pattern, optimal spacing be damned. Of course, optimality of other aspects of the sensory system, such as the biomechanical characteristics of the individual hairs investigated by Bathelier et al. [38], may be equally or even more important than the inter-hair spacing that we investigated here with respect to driving the overall system performance toward functional optimality. This “non-optimal functionality” for sensor spacing in this system could be examined through simulations: could we derive a hair array pattern, different than the actual observed pattern that would yield a higher level of performance against some quantitative functional metric that would not be achievable with the simple developmental model? Such a result would suggest a different perspective on the role played by natural selection in optimizing sensory system functionality: any targets for optimization would have to be in the neural circuitry downstream of the receptors, and “optimization” would then be conceptualized as a mitigation of the inherent limitation(s) of the sensory array structure imposed by developmental mechanisms.

The model presented here is, in essence, a quantitative formulation of earlier hypotheses using the formalism of a mathematical model, along with a demonstration of the plausibility of the simplest possible biological implementation. How might these hypotheses and predictions be tested experimentally? The most direct validation of the core hypothesis would be the identification of two or more chemical morphogen systems which are necessary for the development of a normal patterning of cercal filiform receptor hairs in this system. However, a direct experimental test of our specific prediction that the minimal configuration of only two independent morphogen systems is sufficient for normal development of the receptor pattern is more problematic: tests of “necessity” are more straightforward and definitive than are tests for “sufficiency”. It would need to be demonstrated experimentally that the two organizational aspects of the sensor arrays described in our model could be perturbed independently by manipulation of the minimal number of chemical morphogens represented in our model equations, but by no more than that minimal number. This, of course, is a problem faced by all such studies.

### Limitations of Our Analysis

In this study, we have focused on a consideration of the aspects of the receptor hair patterns that are highly conserved between different crickets. In doing so, we have excluded from our consideration the substantial degree of inter-animal variation in the hair pattern that we documented in a recent paper [2]. An underlying assumption is that the invariant global aspects (discussed in relation to figures 4, 5 and S1) are the ones under selective pressure, and most relevant from functional standpoints. Indeed, the variation in patterns observed experimentally is very localized, and can be conceptualized as being similar to the variation between different fingerprints: all fingerprints look globally similar, but are actually unique at the individual level. The smaller the scale of characterization, the greater the variance. This begs a very important question: does the observed degree of inter-animal variance in aspects of the patterns that we chose to ignore have a functional/behavioral significance? It is possible that the inter-animal variability might be important from some behavioral standpoint? E.g., perhaps the inter-animal variation confers some degree of randomness (or, at minimum, an inter-animal variation) to the behavioral escape responses of a population of crickets, thereby conferring some selective advantage to that group. Alternatively, it could be the case that all of the different patterns we observe across different animals reflect the natural functioning of the morphogen-based developmental mechanisms we have modeled, operating throughout the series of molts between instars in the presence of other “noisy processes” that perturb any individual pattern from some canonical pattern (e.g., different quantities and qualities of food sources, damage to sites on the cerci, or baseline intrinsic “noisiness” in the morphogen systems). Discrimination between these different possibilities will require, at a minimum, additional behavioral analyses of crickets with different array patterns, as well as fluid-dynamics modeling of the functional characteristics of different typical configurations within the normal range of variability.

## Supporting Information

Figure S1 **Ripley’s L function for 3 different cerci and a model result. The circular window diameter is in mm.** The experimental data is shown using colored lines (red, green and blue), and the model result is shown with a black line. While there is variability between the experimental data sets, the overall spatial distribution of the 300 hairs shows a consistent level of segregation across all three experimental cerci.
(TIF)

Figure S2 **Numerical solution to the Turing reaction-diffusion problem given by equation (S1).** The equation was solved using the finite element method. The color scale shows the value of the dimensionless concentration of U, which is the long range inhibitor in the model, plotted onto the surface of a conical structure representing a segment of a cricket cercus. The segment shown here is 0.5 cm in length, and is solved using parameters that generate approximately 300 hairs.
(TIF)

Supplemental Material S1. (DOCX)
