# Supplementary figures and images for: A Model of Filiform Hair Distribution on the Cricket Cercus
Source: PLoS One. 2012 Oct 4;7(10):e46588. doi: 10.1371/journal.pone.0046588 (PMC3464291; doi:10.1371/journal.pone.0046588)

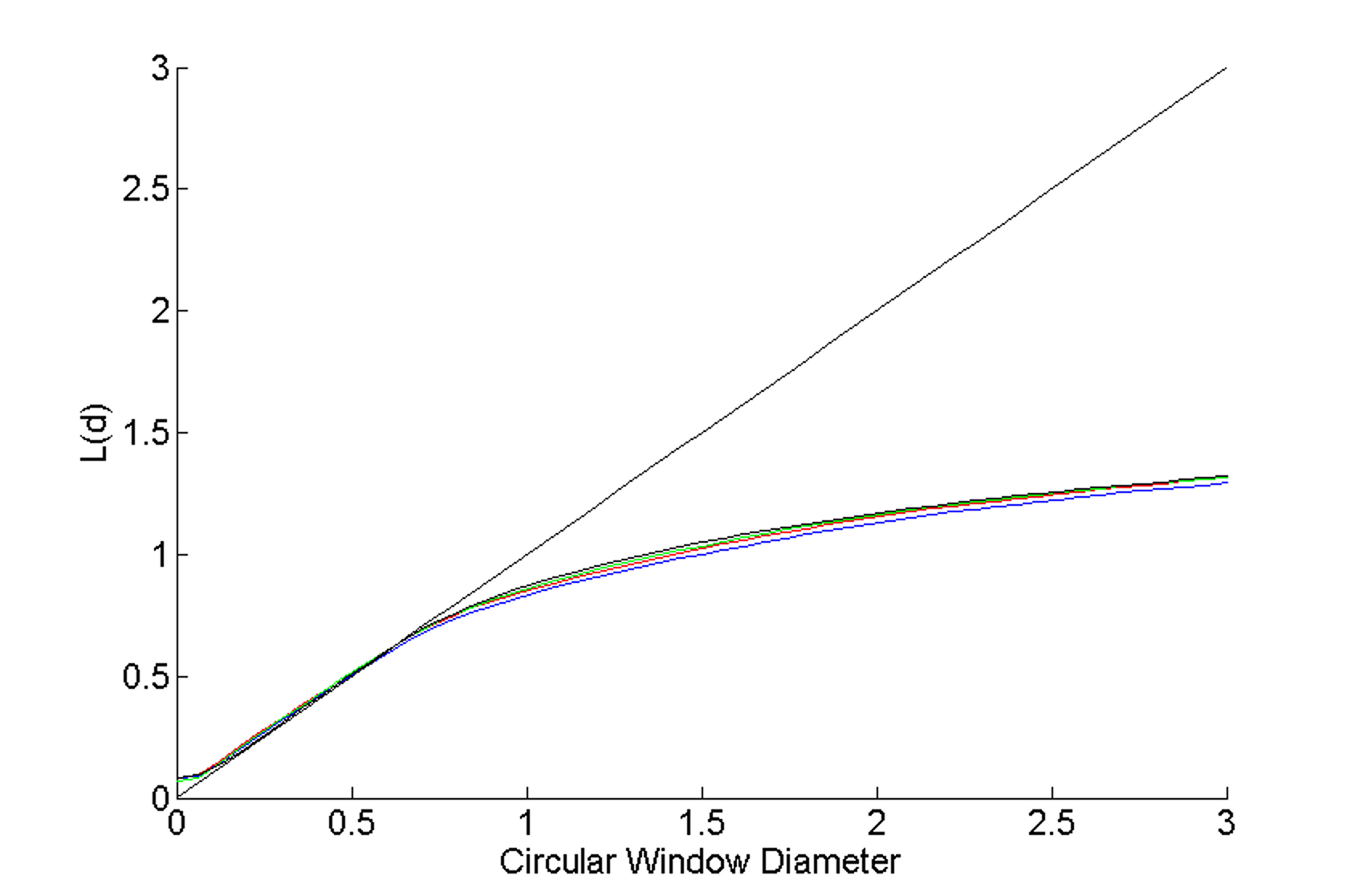

Supplement: Figure S1 — Ripley’s L function for 3 different cerci and a model result. The circular window diameter is in mm. The experimental data is shown using colored lines (red, green and blue), and the model result is shown with a black line. While there is variability between the experimental data sets, the overall spatial distribution of the 300 hairs shows a consistent level of segregation across all three experimental cerci. (TIF) [file pone.0046588.s001.tif]

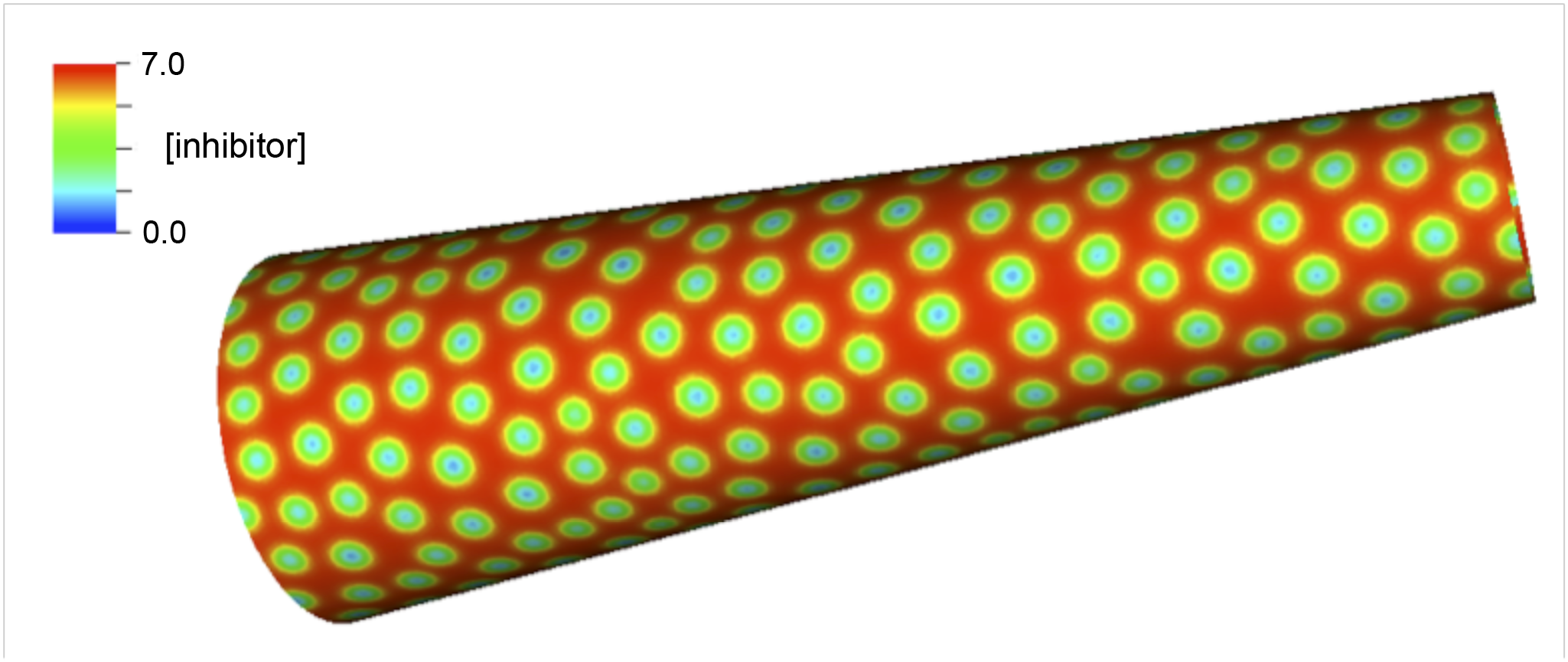

Supplement: Figure S2 — Numerical solution to the Turing reaction-diffusion problem given by equation (S1). The equation was solved using the finite element method. The color scale shows the value of the dimensionless concentration of U, which is the long range inhibitor in the model, plotted onto the surface of a conical structure representing a segment of a cricket cercus. The segment shown here is 0.5 cm in length, and is solved using parameters that generate approximately 300 hairs. (TIF) [file pone.0046588.s002.tif]
