# Supplementary material for: A Model of Filiform Hair Distribution on the Cricket Cercus
Source: PLoS One. 2012 Oct 4;7(10):e46588. doi: 10.1371/journal.pone.0046588 (PMC3464291; doi:10.1371/journal.pone.0046588)
Supplement: Supplemental Material S1. — (DOCX) [file pone.0046588.s003.docx]

### Supplemental Material

**Spatial Autocorrelation Analysis Variability**

The spatial distribution of sensory hairs was quantified in the main paper using Ripley’s L function. Figure S1 shows Ripley’s L function for three different experimental data sets taken from three different cerci. The experimental data is plotted with colored lines, and the model result is shown using a black line. The level of variability between the three data sets is very small and difficult to see visually because the data points are close to each other. Multiple data sets were not shown in Figure 5 in the main paper because of the overlap of the data points.

**Turing Reaction-Diffusion Model**

The mathematical model predicting the distribution and alignment of sensory hairs on the cricket cercus in the main paper is based on a linear combination of solutions to the linear diffusion problem and a Monte Carlo minimization algorithm. An alternative to this approach is a Turing reaction-diffusion (RD) model that is commonly used to predicted biological patterns. Here we present a reaction-diffusion model that predicts the spatial distribution of filiform hairs on the cricket cercus but not the hair alignment directions.

The Turing model is the best known theoretical model used to explain pattern formation in biological systems [[1](#_ENREF_1),[2](#_ENREF_2)]. This model is based on two interacting elements (e.g., two interacting proteins). The first element in the system is responsible for short-range, positive feedback, which causes local concentrations to grow. The second element is responsible for long-range negative feedback, which causes a decrease in the concentration of the first element beyond the immediate, locally elevated concentration. Under certain physiologically reasonable conditions, this system provides a stationary pattern that is qualitatively independent of the initial element concentrations. Further, these interacting elements need not be limited to molecules; cellular signaling circuits can also generate stationary patters. To illustrate the application of the Turing model to the cricket cercus, the following dimensionless system was solved using a finite element discretization:

$\begin{matrix} \frac{\partial U}{\partial t}=F(U,V)-0.03U+0.01\nabla^{2}U \\ 0\leq F(U,V)=0.08U-0.08V+0.15\leq0.2 \\ \frac{\partial U}{\partial t}=G(U,V)-0.08V+0.25\nabla^{2}V \\ 0\leq G(U,V)=0.1U-0.15\leq0.5 \end{matrix}$ (S1)

where U is the dimensionless concentration of the first, low-diffusion rate element and V is the dimensionless concentration of the second, higher diffusion rate inhibitory element. The result of solving these equations on a conical domain with an aspect ratio that is consistent with the cricket cercus is shown in Figure S2. The spot pattern is consistent with the pattern of filiform hair sockets on the cricket cercus. The model given above only accounts for the distribution of hair sockets and does not account for other aspects of the patterns observed on the cricket cercus. For example, this Turing model was not intended, nor set up, to account for the directionality of the hair movements shown in Figure 2. These bands are almost certainly caused by a second morphogen (or chemical signal), which could be modeled using a second Turing model, but would need to be in addition to the first set of Turing equations above.

The standard approach of solving the Turing reaction-diffusion equations with a numerical approximation technique, such as the finite element or finite volume methods, has a high computational cost relative to the approach we used in our study. If N is the number of hairs (or spots), we expect the number of discrete unknowns in a Turing model to scale as O(2N^2^) in 2-dimensions. This is because the discretization grid must be refined to resolve the additional sockets, and the overall cost of solving the discrete problem will be roughly O(2N^4^) for an iterative linear problem solver (e.g., GMRES algorithm) and O(2N^6^) for a direct solver (e.g., LU decomposition algorithm). This contrasts with our approach, which scales as O(N^2^), where N is the number of hairs.

A second significant difference between a standard Turing model and our approach is that our model is discrete and stochastic. With a Turing model, the final steady-state pattern is fixed; e,g, if the simulations are run multiple times with the same initial conditions, all simulations will converge to the same final configuration. Furthermore, the final pattern does not contain the occasional random variations in inter-hair spacing seen in actual cricket cercus specimens. Careful observation of Figure 2 in the main paper shows many instances where two sockets are very close to each other, and/or the directionality of nearby hairs is very different. Neither of these conditions would be obtained through a standard Turing reaction-diffusion model. Our approach does produce randomness of these types. We do not argue that the more realistic pattern characteristics generated by our modeling approach are necessarily due to some aspect of our model that is more “biologically realistic” than a Turing model in some fundamental mechanistic sense. Rather, the precise nature of the stochastic pattern characteristics emerges from the nature of the algorithm we use.

**References**

1. Kondo S, Miura T (2010) Reaction-Diffusion Model as a Framework for Understanding Biological Pattern Formation. Science 329: 1616-1620.

2. Turing AM (1990) The Chemical Basis of Morphogenesis (Reprinted from Philosophical Transactions of the Royal Society (Part B), Vol 237, Pg 37-72, 1953). Bulletin of Mathematical Biology 52: 153-197.

**Supporting Information Legends**

**Figure S1. Ripley’s L function for 3 different cerci and a model result. The circular window diameter is in mm.** The experimental data is shown using colored lines (red, green and blue), and the model result is shown with a black line. While there is variability between the experimental data sets, the overall spatial distribution of the 300 hairs shows a consistent level of segregation across all three experimental cerci.

**Figure S2. Numerical solution to the Turing reaction-diffusion problem given by equation (S1).** The equation was solved using the finite element method. The color scale shows the value of the dimensionless concentration of U, which is the long range inhibitor in the model, plotted onto the surface of a conical structure representing a segment of a cricket cercus. The segment shown here is 0.5 cm in length, and is solved using parameters that generate approximately 300 hairs.
